# Barriers to adopting a healthy lifestyle: insight from postpartum women

**DOI:** 10.1186/1756-0500-2-161

**Published:** 2009-08-17

**Authors:** Lori Carter-Edwards, Truls Østbye, Lori A Bastian, Kimberly SH Yarnall, Katrina M Krause, Tia-Jane'l Simmons

**Affiliations:** 1 Department of Community and Family Medicine, Duke University Medical Center, Durham, NC, USA; 2 Department of Internal Medicine, Duke University Medical Center, Durham, NC, USA

## Abstract

**Background:**

Postpartum weight retention can contribute to obesity. There may be unique barriers to weight loss in this period.

**Findings:**

Cases are presented for three postpartum women who declined to participate in a postpartum weight loss intervention.

Despite their desire to engage in healthier behaviors, or partake in an intervention uniquely designed to promote healthy lifestyles for postpartum women, some find it too difficult to make such commitments. Barriers women face in adopting a healthier lifestyle in this period include 1) time availability; 2) prioritizing other competing life responsibilities above their own health; 3) support from family members, friends, and/or co-workers; and 4) lack of flexibility in the intervention structure. These illustrations describe their perspectives in the context of life balance, perceived health, and support, and reflect the multi-dimensional nature of their lives during the life cycle change of the postpartum period.

**Conclusion:**

Postpartum women face difficult and complex challenges to prioritizing their health and their weight management.

## Introduction

The postpartum period is a transitional phase involving lifestyle and body weight changes. Postpartum weight retention is common [1,2] and can have long-term impacts on health [2,3]. This weight retention is as much associated with modifiable lifestyle factors, including diet and physical activity, as with characteristics such as prepregnancy body weight [4-6]. Thus, behavioral interventions to reduce postpartum weight retention are warranted.

Participating in such programs, however, can be a challenge. Only two community-based randomized trials to reduce postpartum weight retention have been conducted [7], in which the drop-out rates were 27% [8] and 40% [9], respectively. Participation in such interventions may be complicated by factors unique to this period, including difficulty and lack of time to attend structured activities while caring for infants [10]. However, data collected directly from postpartum women are lacking.

Through semi-structured, in-depth personal interviews, we explored barriers faced by women who refused participation in a postpartum weight loss lifestyle intervention. Understanding factors that impact participation in such programs is critical to designing appropriate and successful interventions, and the following cases illustrate reasons why postpartum women interested in losing weight declined to participate.

## The active mothers postpartum study

Active Mothers Postpartum (AMP) was a randomized controlled behavioral intervention trial to promote postpartum weight loss via sustainable lifestyle changes, including decreased calorie intake and increased physical activity [11]. Women were contacted for screening at 4 weeks postpartum, to facilitate an enrollment visit at 6 weeks postpartum. The intervention consisted of 10 physical activity classes and 8 nutrition education classes over a 9 month period. Classes were offered multiple times a week, including nights and weekends. Four hundred fifty overweight and obese postpartum women in the Triangle area of North Carolina were randomized to the trial from September 2004 to March 2006.

Women were identified for the present case studies during the last stages of recruitment (October 2005 – March 2006). Women in this period who refused to complete telephone screening for the AMP trial (n = 48) but remained engaged long enough to have the study explained to them in full (n = 10) were asked about their willingness to participate in a one-time interview regarding barriers to participation. Four women agreed to be contacted by phone to be interviewed, and three were successfully reached to schedule an interview time. These three women completed an informed consent and brief survey of demographics, support network size, parity, and transportation.

Semi-structured interviews with each woman were audio-taped and subsequently transcribed [12]. The one-hour interviews included questions on: roles and responsibilities since the newborn's birth; support provided by others; and what it would take to participate in a lifestyle modification program. Explored were barriers to healthy lifestyle changes and motivating forces that would have encouraged them to engage in the intervention. Transcripts were independently coded for primary themes by the first three authors (LCE, TØ and LAB) and reviewed for concordance. This qualitative study was approved by the Duke Institutional Review Board.

## Ms. X – "You just can't do it all"

Ms. X, a Caucasian woman, age 41 years, is a mother of five children whose fifth child is a 5-month old infant. Her husband is a pastor of a church. Ms. X, who is college educated, does not work outside the home and homeschools her children. The one health behavior change she would like to make is to lose weight. She used to walk with a partner, but does not have the time that she used to. Given her array of responsibilities, Ms. X is tired much of the time and gets little rest. In terms of life priorities, her spirituality comes first, followed by her relationship with her husband and her children. Her primary concern is her responsibility to her newborn, within the context of her other children and family tasks. Since the birth of this child, the family has re-prioritized their activities by limiting the amount of time spent on social events outside of the home. Her husband helps more with the household chores, and the neighboring mother-in-law has become involved with the children's education.

Ms. X felt that the costs in geographic location, time commitment, effort, and general inconvenience were not worth the overall benefits of participation in the AMP study. She felt that she would have to perceive her health issues to be more serious to be encouraged to change her lifestyle. Ms. X felt that social support, such as good friends in the program with her, would motivate her to participate.

## Ms. Y – "My health, it kind of takes a back burner"

Ms. Y is a 25-year-old Caucasian stay-at-home mother of two, 3 years old and 5 months old, who lives on the outskirts of the city. The one health behavior change she would like to make is to eat regularly instead of waiting to eat a large meal in the evening. She mentioned difficulty finding personal time to eat during the day while caring for her children's needs, and financial constraints in affording healthier food. She eats quick, healthy snacks, and feeds her children healthy food while she nurses her newborn; however, she also purchases and eats less nutritious snacks out of convenience. She believes that she should only be living now for her children, not for herself, and that her children's welfare should take precedence over her taking care of her own health.

Ms. Y did not participate in the AMP Study because she would only have been permitted to bring her newborn and not her other two children to the program classes. She is used to taking her children with her everywhere she goes. She keeps her children close because her mother did not spend a lot of time with her during her childhood, and because she lost a son 8 1/2 months in gestation. She may have participated, if she had the option of attending a limited number of classes.

## Ms. Z – "It's non-stop all day"

Ms. Z, 23 years of age, is a Caucasian full-time employee at a pharmacy and mother of three young children, 5 years old, 3 years old, and 2 months old. She is married and lives on the outskirts of the city. The one health behavior change she would like to make is to exercise more, since it makes her feel better. Ms. Z does not understand why she is often tired, yet regularly gets at least eight hours of sleep at night. She is constantly in motion at home and on her job, which requires her to be on her feet. She believes her husband does not understand her need for time to herself because he works 10-hour shifts. Although her husband does not initiate support, if she asks him to do something specific he will fulfill her request. However, he has difficulty handling more than one child at a time.

She wanted to be involved in the AMP study, but the timing of classes, her work schedule and that of her husband, and her mother's varied availability to watch the children after work hours, prohibited her commitment.

All three women described their responsibilities to their families and expressed the impact the increased efforts with the newborn added to their already busy schedules. There are several common themes in the barriers these women faced that may also be common for other postpartum women.

### Limited time for personal health

Ms. X perceived that a program for exercise and behavior changes was an addition to her already packed schedule. The fact that she had already reduced family activities outside the home, and eliminated her own walking program to relieve the time crunch, points to the time barrier many postpartum women experience. Ms.Y found it difficult to find time to eat well during the day, and viewed things that took her away from her children as extra time commitments. Ms. Z knows exercise makes her feel better but found that the classes did not fit into her or her spouse's work schedules. All three women required the interviewer to call after 9:00 pm, when family demands were lower. Clearly, the sense of inadequate time for an intervention aimed at improving health was a significant barrier to participation.

### Prioritizing one's children above oneself

Ms. Y felt that her health was not as important as the welfare of her children. Though she perceived herself as overweight, she put her children's needs and her need to spend time with them above her own health concerns. Ms. X shared this barrier, conveying that her health was not poor enough to be actively involved in lifestyle changes. Both Ms. X and Ms. Y would make a commitment to engage in improved health behaviors if they perceived that their health was life-threatening or had immediate, noticeably negative consequences. Thus, they would need to feel threatened by their current behaviors and believe that behavior change would be beneficial with a cost that is acceptable to them [13,14]. Ms. Z reports being tired despite adequate sleep and improved feeling of well being when she exercises, but fails to make the connection that addressing her health and fitness will improve her fatigue. All three women express that at this point in their lives their personal health was not top priority. As multicaregivers, they have a myriad of responsibilities to others to the point where they typically put themselves last.

### Social support – both positive and negative effects

Social support was considered important to all three women. Ms. Z expressed the need for a stronger social network and did not have many social contacts outside of her employment. She did not receive strong support from her husband, and felt uncomfortable asking him to care for more than one child at a time. At work she felt guilty when she missed work due to children's illness, though she appreciated the support when others traded shifts with her. She would have liked to participate in AMP but lacked the social support at home and work. Ms. Y had little contact with adults throughout the day, but received emotional support from telephoning her friend and periodically joining her friend in taking their children to the mall, park, natural science center, or for a walk. Ms. X received reciprocal support from church members by exchanging meals for child care; however, she must often accept unhealthy food in these exchanges. Thus, Ms. X's church members provide needed instrumental support, but that support also enables unhealthy eating behaviors. As postpartum mothers, all three women's support networks could be important motivators to engage in healthy lifestyle behaviors, or barriers to their daily functioning [15].

### Logistics

For all three women, geography was a barrier to participation. Since time to meet their multitude of priorities is limited, particularly in the presence of a newborn, adding time to drive to the classes, coupled with the unavailability of care for additional children, made it difficult to make a commitment to participate.

## Conclusion

Barriers postpartum women face in adopting healthier lifestyles include child care and time management issues; perceptions of their health in the context of competing responsibilities; support from family members, friends, and/or co-workers; and lack of flexibility in the intervention structure, including distance. Each mother has multiple responsibilities to those around them, even to the expense of improving their health [16].

Interventions to address lifestyle behaviors of postpartum women must take these multiple inter-related barriers into account. It does appear that for the participants in this research, the demands of caring for an infant made prioritizing their own health or attending activities outside the home too overwhelming of a commitment [10]. Social support from family and friends is a potential motivator to engage in lifestyle behavior changes, and the form of that support (i.e., meeting together to walk, talking on the phone) may be useful strategies for sustainable behavior change [17,18]. Although interventions may not be able to address every barrier, successful behavior modification may depend on identifying women's priorities and designing interventions that effectively incorporate them.

## Competing interests

The authors declare that they have no competing interests.

## Authors' contributions

LCE conceived of and executed the study, conducted the interviews, and wrote the manuscript. TØ and LAB assisted in the design of the study, interpretation of the data, and revision of the manuscript. KSHY, KMK and TJS assisted in interpretation of the data and revision of the manuscript. All authors approved of the final manuscript.
